# Para-aortic lymphadenectomy in advanced stage cervical cancer, a protocol for comparing safety, feasibility and diagnostic accuracy of surgical staging versus PET-CT; PALDISC trial

**DOI:** 10.1186/s40814-017-0218-8

**Published:** 2018-01-04

**Authors:** Casper Tax, Karin Abbink, Maroeska M. Rovers, Ruud L. M. Bekkers, Petra L. M. Zusterzeel

**Affiliations:** 1 Department of Operating Rooms 715, Radboud University Medical Centre, Radboudumc Institute for Health Sciences, P.O. Box 9101, 6500 HB Nijmegen, the Netherlands; 2 Department of Gynaecology, Radboud University Medical Centre, Radboudumc Institute for Health Sciences, P.O. Box 9101, 6500 HB Nijmegen, the Netherlands; 3 Department of Health Evidence, Radboud University Medical Centre, Radboudumc Institute for Health Sciences, P.O. Box 9101, 6500 HB Nijmegen, the Netherlands

**Keywords:** Diagnostic accuracy, PET-CT, Advanced stage cervical cancer, Para-aortic lymph node metastases, Para-aortic lymphadenectomy, Safety, Feasibility, Surgical staging

## Abstract

**Background:**

Currently, a PET-CT is used to assess the need for extended field radiotherapy of para-aortic lymph nodes (PALN) in International Federation of Gynaecology and Obstetrics (FIGO) stage IB2, IIA2–IVA (locally advanced stage) cervical cancer. A small study established a sensitivity and specificity estimate for PALN metastases of 50% (95% CI; 7–93%) and 83% (95% CI; 52–98%), respectively. Surgical staging of PALN may lead to a higher diagnostic accuracy. However, surgical staging of para-aortic lymph nodes in locally advanced stage cervical cancer is not common practice. Therefore, a phase 2 randomised controlled trial is needed to assess its safety and feasibility.

**Methods/design:**

In addition to standard imaging (MRI or CT scan) with PET-CT, 30 adult women with FIGO stage IB2, IIA2–IVA cervical cancer will be randomised to receive either surgical staging or usual PET-CT staging. Administering extended field radiotherapy will be based on lymphadenectomy results for the intervention group and on the PET-CT results for the control group. Follow-up visits at 0, 3, 6, 9 and 12 months will assess health-related quality of life and progression-free survival.

Primary safety and feasibility outcomes of surgical staging will be assessed by calculating means with 95% confidence intervals for duration of surgery, number of complications, blood loss, nodal yield after para-aortic lymphadenectomy and treatment delay due to surgical staging. Secondary patient-centred outcomes on quality of life and first year survival will be documented and compared between the two groups. Estimates of sensitivity, specificity and negative and positive predictive values of MRI, PET-CT and surgical staging will be presented with 95% CI.. All analysis will be performed according to the intention to treat principle.

**Discussion:**

This study will assess safety and feasibility, expressed as the number and severity of complications, effect on quality of life and the treatment delay due to surgically staging para-aortic lymph nodes in locally advanced cervical cancer. It will provide insight in the diagnostic accuracy of the PET-CT and detection rate of missed (micro)metastases due to surgical staging. This information will be used to assess the necessity for a phase 3 study on the diagnostic accuracy of the PET-CT and surgical staging. If a phase 3 study is deemed necessary, current data can be used for sample size calculation of such a phase 3 study.

**Trial registration:**

Nederlands Trial Register/Dutch Trial Registry (www.trialregister.nl), NTR4922. Registered on 24 November 2014.

## Background

Cervical cancer is fairly rare in the Netherlands, with an incidence of approximately 700 new diagnoses each year or approximately 8.3 new diagnoses per 100,000 women per year in the Netherlands [1]. Staging is based on the International Federation of Gynaecology and Obstetrics (FIGO) staging system for cervical cancer. It uses clinical features and becomes increasingly inaccurate as the disease progresses, with accurate staging failure rates rising from 25% in FIGO stage I and II to 65–90% in stage IIIB [2]. The lymph node status of pelvic and para-aortic lymph nodes is, besides tumour stage, the most important prognostic factor for disease recurrence in cervical cancer [3]. Failure to detect metastases in these lymph nodes may lead to suboptimal treatment. Para-aortic lymph node (PALN) metastases are quite common in FIGO IIA patients with positive pelvic lymph nodes (16%), whereas solitary PALN are rare (0.8%) [4]. PALN metastases affect around 20% of all stage IB2–IVA, although other studies claim considerably higher rates up to 40% [4–7].

Patients with advanced stage cervical cancer (FIGO stage IB2, IIA2–IVA) are primarily treated with pelvic radiotherapy and concomitant chemotherapy. Para-aortic lymph node (PALN) status and a subsequent need for extended field radiotherapy is assessed using a positron emission tomography (PET) combined with computed tomography (CT) scan (PET-CT). PET-CT provides a higher sensitivity and specificity than magnetic resonance imaging (MRI) [8, 9]. However, for detecting PALN metastasis, PET-CT seems to have a sensitivity and specificity of 50% (95% CI; 7–93%) and 83% (95% CI; 52–98%), respectively [10]. This uncertainty and these broad confidence intervals are due to the fact that only one small study (*n* = 16) explicitly assessed the diagnostic accuracy of the PET-CT. So far, this study is the only study that compared the PET-CT results of para-aortic lymph nodes in cervical cancer to the gold standard of histology. More evidence on diagnostic accuracy of PET-CT in detecting PALN metastases is needed. Some intervention studies provide a part of these data as they include surgical staging and the PET-CT in advanced stage cervical cancer, such as the LILACS trial [11]. However, in the LILACS trial, surgical staging is only performed in PET-CT negative patients and not in PET-CT positive patients. This may be due to the fact that the PET-CT seems to have a sensitivity of 50% and specificity of 83%, thus possibly being false negative in half of all patients with PALN metastases while being false positive in 17% of all patients without PALN metastases, or due to the assumption that the specificity and positive predictive value of the PET-CT are already acceptable. Regardless, although missing a PALN metastases due to a false negative result may be more detrimental to survival than ‘overtreating’ patients due to a false positive result, the radiotherapy-induced complications can also have a negative impact on the quality of life of patients. In addition, the confidence interval surrounding the specificity of the PET-CT ranges from 52 to 98%, leading to a large uncertainty. As such, more evidence on both the sensitivity and specificity of the PET-CT is warranted..

Recent studies show that surgical staging not only validates the imaging results, but may also lead to a treatment modification in 20–40% of the patients with locally advanced cervical cancer compared to PET-CT results [2, 12–16]. Surgical staging, i.e. removing the para-aortic lymph nodes by performing a para-aortic lymphadenectomy, leads to a gain in diagnostic accuracy by allowing adequate histological evaluation of the retrieved lymph nodes. Additionally, the resection of enlarged positive lymph nodes may provide therapeutic benefit [2, 15–18]. Whether this is due to removing the bulk of the disease or ensuring the radiation field includes confirmed nodal disease, or both, is unclear.

The proportion of treatment modifications may depend on both the diagnostic accuracy of the PET-CT and a clinician’s experience with interpreting the PET-CT results and performing the surgical staging. The validity of detecting metastasis with surgical staging compared to standard diagnostics remains to be elucidated. Currently, PET-CT is the gold standard in the Netherlands when there is a suspicion of para-aortic lymph node metastases based on either clinical data, MRI or CT scan. In addition, surgical staging of PALN in locally advanced stage cervical cancer is not common practice. Therefore, a phase 2 trial to assess safety and feasibility is warranted.

The PALDISC (*P*ara-*A*ortic *L*ymphadenectomy *I*n advanced stage *C*ervical cancer) trial will assess safety and feasibility of surgical staging in locally advanced cervical cancer in the Netherlands. It will provide insight in diagnostic accuracy of the PET-CT and detection rate of missed (micro)metastases due to surgical staging. This information will be used to assess the necessity for a phase 3 study on the diagnostic accuracy of the PET-CT and surgical staging. If a phase 3 study is deemed necessary, current data can be used for the sample size calculations of such a phase 3 study.

## Methods/design

**Fig. 1:**
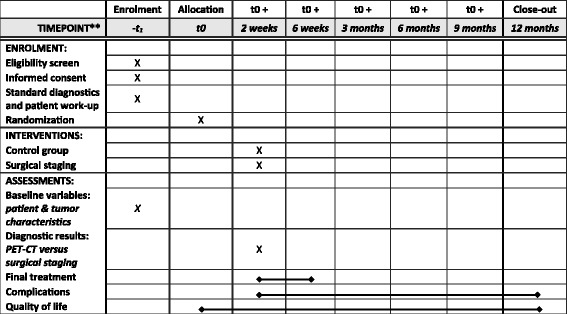
SPIRIT flow diagram

This study is a multi-centre randomised controlled phase 2 trial with 1 year of follow-up. The study is currently being conducted at the Radboudumc Nijmegen and Catharina Hospital Eindhoven in the Netherlands. A general overview of the study is presented in a SPIRIT flow diagram (Fig. 1).

### Inclusion criteria

Women (aged ≥ 18 years) with FIGO IB2, IIA2, IIB, IIIA, IIIB and IVA cervical cancer from general and specialised hospitals for gynaecologic care in the Netherlands are eligible for randomization after meeting the following criteria:

1. Age of 18 years or above;
2. Histologically confirmed squamous cell, adenosquamous or adenocarcinoma of the cervix;
3. FIGO stage IB2, IIA2, IIB, IIIA, IIIB and IVA, staging performed by examination under anaesthesia;
4. Fit for surgery;
5. WHO-performance 0-2;
6. WBC > 3.0 × 109/L, platelets > 100 × 109/L, creatinine clearance > 60 ml/min;
7. Chest CT or X-ray, abdominal MRI or CT scan and PET-CT with no evidence of distant metastasis (PET positive > 2 cm);
8. Written informed consent.

### Exclusion criteria

A potential subject who meets any of the following criteria will be excluded from participation in this study:

- Previous malignancy (except for non-melanoma skin cancer)
- Prior retroperitoneal surgery
- Previous pelvic or abdominal radiotherapy
- Upper abdominal intraperitoneal disease or evidence of ovarian metastasis
- Evidence of distant metastasis on imaging or physical examination
- Bulky para-aortic lymph nodes > 2 cm
- Pregnancy
- Otherwise unfit for surgery

### Randomisation, blinding and treatment allocation

After providing informed consent, all patients will be randomised by the enrolling clinician using the randomization program provided by Castor EDC. Patients will be randomised equally between surgical staging and standard treatment using a permuted block design per centre; the allocation sequence itself is concealed from the patient, investigator and clinician [19]. Blinding of the patient, investigator and/or observer from the allocation result is not possible.

### Intervention, surgical procedure and histological analysis

**Fig. 2:**
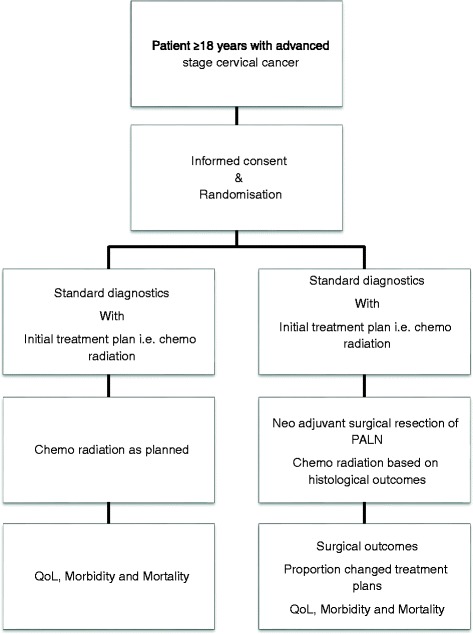
Patient flow

Figure 2 shows the patient flow. Patients in the intervention arm will receive surgical staging of PALN, besides the regular treatment and imaging (including PET-CT) of locally advanced stage cervical cancer. This will be a laparotomic, laparoscopic or robot-assisted node by node para-aortic lymphadenectomy from the common iliac bifurcation up to the left renal vein, performed by an experienced team of retroperitoneal surgeons. In case of a laparoscopic or robotic procedure, the surgeon may choose between trans- or peritoneal approach. All open procedures should be based on a retroperitoneal approach due to reported increased morbidity [15, 20–22]. A sentinel node procedure, as well as a debulking of bulky pelvic nodes, and an ovarian displacement to avoid radiotherapy on an ovary may be performed optionally.

Histological analysis of PALN will be performed node by node and includes all normal procedures with an additional assessment on lymph node yield, node diameter and diameter of micro metastases. Patients with metastatic PALN on histological analysis will receive extended field radiation therapy with concurrent chemotherapy and optional adjuvant chemotherapy. To ensure a comprehensive assessment of the primary tumour, nodal sites and potential metastatic sites, a physical examination including a rectovaginal examination under anaesthesia and radiology including a chest CT or X-ray, abdominal MRI and PET-CT scan are performed. All PET-CT-detected para-aortic lymph nodes will be described and registered per region. In the surgical staging group, all PALNs will be excised, described and registered per region in order to correlate radiological, surgical and histological findings.

### Control group

Patients in the control group will receive treatment according to the current guidelines, i.e. treatment with regards to PALN status will be based on PET-CT.

### Radiotherapy and adjuvant chemotherapy

All patients will be treated with both external beam radiotherapy (EBRT) and brachytherapy. The choice for applying extended field radiotherapy will be based on surgical staging or PET-CT, according to the results of the study arm. Concomitant chemotherapy will consist of 5 cycles of weekly cisplatin (40 mg/m2), coinciding the first dose of chemotherapy with the first dose of radiotherapy.

### Main study parameters and analysis

The main study parameters are the safety and feasibility of para-aortic surgical staging. The feasibility will be assessed by registering a proportion of included patients amongst all eligible patients. We will assess the safety by calculating means with 95% confidence intervals (CI) for length of surgery, number of complications per Clavien-Dindo category (Appendix 1), amount of blood loss and number of adverse events which will be reported using the Serious Adverse Event Report (SAE) form (Appendix 2) [23]. Also, mean nodal yield with 95% CI after para-aortic lymphadenectomy will be presented and compared to results found in other studies in order to assess completeness of lymphadenectomy.

### Secondary study parameters and analysis

Although this is a phase 2 trial, we will also register secondary outcome parameters such as (progression-free) survival and quality of life as they are also aspects of safety. The incidence of advanced stage cervical cancer is quite low in the Netherlands. Including these secondary parameters will make it possible to enter these patients into a subsequent phase 3 trial, if the current PALDISC study shows that such a phase 3 study is indeed necessary. Decision on the necessity of a phase 3 trial will depend on whether surgical staging of PALN in advanced stage cervical cancer is deemed safe, feasible and necessary in comparison with the performance of current PET-CT imaging.

The proportion of changed initial treatment plans and diameter of metastases will be documented and presented. Data on sensitivity, specificity and negative and positive predictive values of MRI, PET-CT and surgical staging will be documented in 2 × 2 tables; estimates will be provided with 95% CI.

Time to treatment will be measured in days from the day of randomization until start of chemo radiation. Difference in treatment delay between the arms, due to surgical intervention and histological analyses, will be presented using a Kaplan-Meier curve with the corresponding medians and 95% CI. Rate differences and 95% CI for mortality and morbidity during follow-up will be calculated and presented in a table.

The analysis of progression-free survival and overall survival will be based on and presented in a Kaplan-Meier curve with corresponding hazard ratio and 95% CI. Means and range in reported quality of life will be reported using the Dutch versions of the European Organisation for Research and Treatment of Cancer (EORTC) Core Questionnaire, the QLQ-C30 version 3.0 and the supplemental cervical cancer-specific module, the QLQ-CX24 [24, 25]. The difference in quality of life between the arms will be presented as a mean difference with 95% CI.

### Data entry, missing data, loss to follow-up and withdrawal

All data will be analysed on an intention to treat basis. Data entry, coding, security and storage are planned and provided within Castor EDC, including data quality checks such as checks on double data entry, range checks for data values, etc. [19].

Missing data will be imputed with multiple imputation if assumed to be missing at random. Patients lost to follow-up or dropping out will not be replaced for it might indicate problems in feasibility.

Subjects can leave the study at any time for any reason if they wish to do so without any consequences. The reason will be documented on the case report form. Patients who have withdrawn themselves from the study will receive treatment and follow-up according to national protocol.

### Sample size calculation, data monitoring and premature termination of the study

We will include 15 patients in each arm for this phase II study. There will be no formal data-safety monitoring board due to the relatively small number of patients (*n* = 15) in the intervention arm. However, due to the nature of this intervention and possible risk related to the intervention, we have installed an independent expert to ensure the safety and ethics. After each serious adverse event, which may in any way be related to the para-aortic lymphadenectomy, all inclusions and planned interventions at all centres will be stopped immediately. All principle investigators will converge and decide whether it is ethical to resume inclusion after the serious adverse event has abated or until a stable situation has been reached. All meetings and decisions will be documented. Each conclusion will be submitted to the independent expert for an independent assessment on safety and ethics. The trial will only resume after both the principle investigators and independent expert agree that safety and ethical criteria are being met. An independent monitor will check all centres on informed consent, adherence to the in- and exclusion criteria, reporting of all adverse events and full source document verification. The monitor will audit after the first three included patients and then yearly or after every 10 included patients.

### Recruitment and consent

The rationale, design and aims of the study will be explained to each patient along with the specific information on the respective treatment arms. The principles of registration and the follow-up procedure will be explained, and patients will receive written patient information and will have ample opportunity to ask questions. Written informed consent of the patient is required before inclusion and will be obtained by the clinician.

### Risk assessment

Due to experience with the procedure and known incidence and severity of complications, the risk associated with the intervention is estimated to be moderate. Para-aortic lymphadenectomy is new in cervical cancer but a common procedure for gynaecological oncologists in ovarian and endometrial cancer.

### Ethical considerations

The protocol has been reviewed and approved by the Radboud Medical-Ethical Committee (METC) on the 4th of September 2014 with reference number: 2014/240 and registration number: NL49310.091.14, as well as approved by the Radboudumc Board of Directors on 21st of October 2014 with reference number: RvBI4.5 L737. (supplements in Dutch) The study is being conducted in full conformance with the ethical principles of the Declaration of Helsinki Seoul, 2008, the WMO, the Dutch guidelines ‘richtlijn toetsingsprocedure multicenter-onderzoek’ (active as of 1 January 2001) and ‘good clinical practice’.

### Public disclosure, availability of data and materials and publication policy

Within 1 year after the end of the study, a final study report with the results of the study will be submitted, including any publications/abstracts of the study to the accredited ethical committee. Publications and abstracts will be published according to policy of the Central Committee on Research Involving Human Subjects.

The final trial results will be submitted to a peer-reviewed scientific journal for publishing irrespective of the nature of the results.

## Discussion

The PALDISC trial will assess safety and feasibility of surgical staging in patients with locally advanced cervical cancer in the Netherlands. It will provide insight in diagnostic accuracy of the PET-CT and detection rate of missed (micro)metastases due to surgical staging.

So far, all available evidence regarding the validity and diagnostic accuracy of PET-CT in detecting PALN metastases in locally advanced cervical cancer is based on a study with 16 patients only [10]. This leads to a high uncertainty regarding both sensitivity and specificity, which is represented by the large confidence intervals. As a result, patients may have a high chance on false negative and/or false positive PET-CT results, leading to subsequent under- and overtreatment, respectively. Undertreatment may lead to a deterioration of overall and progression-free survival, while overtreatment may lead to unnecessary radiation. Both have detrimental effects on the quality of life of patients as well as hospital and health care expenses.

To compare the effectiveness and diagnostic accuracy of a PET-CT with surgical staging, a phase 3 randomised controlled trial is the most warranted design. However, as para-aortic lymphadenectomy is not standard of care for cervical cancer patients in the Netherlands, we feel the need to first assess its safety and feasibility.

A major strength of our study is that safety and feasibility of the para-aortic lymphadenectomy is assessed on both subjective (quality of life) and objective (time of treatment delay, blood loss, adverse events, etc.) outcome measures. In addition, data on both sensitivity and specificity should also be considered. Although this study only includes 15 patients, it is almost a doubling of the currently available number of patients in whom PET-CT was compared to gold standard histology (*n* = 16) [10]. Furthermore, these outcomes can be compared to current standard of care while controlling for confounders and selection bias due to the randomization. These strengths will provide vital information for a group of experts in order to make the final decision whether or not a phase 3 trial is deemed safe and necessary.

As for most surgical trials, blinding is not possible. To preclude bias due to this lack of blinding, we have chosen objective outcomes on safety and feasibility as the primary outcome for this trial and the subjective quality of life as a secondary outcome. Combining these results will provide insight in the safety and feasibility of para-aortic lymphadenectomy in advanced stage cervical cancer.

## Trial status

The PALDISC trial is currently cancelled, and further inclusion is on hold due to a too low inclusion rate. Recruitment began in July 2015 and a total of five patients were enrolled by October 2017. This low inclusion rate which has led to the cancellation of the study is mainly due to the fact that the incidence of advanced stage cervical cancer is low in the Netherlands and there was only one actively participating centre. With the publication of this protocol, we do hope to spark the interest of other centres for such a trial. In addition, we would like to open a scientific discussion on the diagnostic accuracy of the PET-CT as there is currently no study that compares PET-CT positive results to gold-standard histology analyses. The specificity of 83% (95% CI; 52–98%) based on only 16 patients implies that possibly 17% of all patients have false positive PET-CT results without PALN metastases. This may lead to misinformed treatment decisions including ‘unnecessary’ extended field radiotherapy with associated comorbidities. As such, more evidence on both the sensitivity and specificity of the PET-CT is warranted.

## Abbreviations

AE: Adverse event
CI: Confidence interval
CT: Computed tomography
EBRT: External beam radiotherapy
EORTC: European Organisation for Research and Treatment of Cancer
FIGO: International Federation of Gynaecology and Obstetrics
METC: Medical-Ethical Committee
MRI: Magnetic resonance imaging
PALN: Para-aortic lymph node
PET: Positron emission tomography
QLQ-C30: Quality of Life Core Questionnaire
QLQ-CX24: Quality of Life Questionnaire cervical cancer specific
WBC: White blood cell
WHO: World Health Organization
WMO: Medical Research Involving Human Subjects Act (in Dutch: Wet Medisch-wetenschappelijk Onderzoek met Mensen
